# Mechanistic Modeling Reveals Adaptive Photosynthetic Strategies of *Pontederia crassipes*: Implications for Aquatic Plant Physiology and Invasion Dynamics

**DOI:** 10.3390/biology14060600

**Published:** 2025-05-25

**Authors:** Lihua Liu, Xiaolong Yang, Piotr Robakowski, Zipiao Ye, Fubiao Wang, Shuangxi Zhou

**Affiliations:** 1 College of Safety Engineering and Emergency Management, Nantong Institute of Technology, Nantong 226002, China; liulihua032007@126.com; 2 School of Life Sciences, Nantong University, Nantong 226019, China; yangxl@ntu.edu.cn; 3 State Key Laboratory of Environmental Chemistry and Ecotoxicology, Research Center for Eco-Environmental Sciences, Chinese Academy of Sciences, Beijing 100085, China; 4 Department of Forestry, Poznan University of Life Sciences, Wojska Polskiego 71E St., 60-625 Poznan, Poland; piotr.robakowski@up.poznan.pl; 5 Math & Physics College, Jinggangshan University, Ji’an 343009, China; wangfubiao@jgsu.edu.cn; 6 Department of Biological Sciences, Macquarie University, Sydney, NSW 2000, Australia

**Keywords:** aquatic macrophytes, photosynthetic plasticity, light stress adaptation, photoprotection, chlorophyll fluorescence, mechanistic modeling

## Abstract

The invasive aquatic macrophyte *Pontederia crassipes* (water hyacinth) exhibits exceptional adaptability across a wide range of light environments, yet the mechanistic basis of its photosynthetic plasticity under both high- and low-light stress remains poorly resolved. This study integrated chlorophyll fluorescence and gas-exchange analyses to evaluate three photosynthetic models—rectangular hyperbola (RH), non-rectangular hyperbola (NRH), and the Ye mechanistic model—in capturing light-response dynamics in *P. crassipes*. The Ye model provided superior accuracy (*R*^2^ > 0.996) in simulating the net photosynthetic rate (*P*_n_) and electron transport rate (*J*), outperforming empirical models that overestimated *P*_nmax_ by 36–46% and *J*_max_ by 1.5–24.7% and failed to predict saturation light intensity. Mechanistic analysis revealed that *P. crassipes* maintains high photosynthetic efficiency in low light (*LUE*_max_ = 0.030 mol mol^−1^ at 200 µmol photons m^−2^ s^−1^) and robust photoprotection under strong light (*NPQ*_max_ = 1.375, PSII efficiency decline), supported by a large photosynthetic pigment pool (9.46 × 10^16^ molecules m^−2^) and high eigen-absorption cross-section (1.91 × 10^−21^ m^2^). Unlike terrestrial plants, its floating leaves experience enhanced irradiance due to water-surface reflection and are decoupled from water limitation via submerged root uptake, enabling flexible stomatal and energy regulation. Distinct thresholds for carboxylation efficiency (*CE*_max_ = 0.085 mol m^−2^ s^−1^) and water-use efficiency (*WUE*_i-max_ = 45.91 μmol mol^−1^ and *WUE*_inst_ = 1.96 μmol mmol^−1^) highlighted its flexible energy management strategies. These results establish the Ye model as a reliable tool for characterizing aquatic photosynthesis and reveal how *P. crassipes* balances light harvesting and dissipation to thrive in fluctuating environments. These resulting insights have implications for both understanding invasiveness and managing eutrophic aquatic systems.

## Simple Summary

Water hyacinth is a floating aquatic plant known for its rapid spread and ability to survive in different environments. However, how it adjusts to both strong sunlight and low light is not fully understood. In this study, we used advanced tools to measure how the plant uses light for photosynthesis and protects itself from light damage. We tested three models that simulate how plants respond to light and found the Ye model, commonly used for terrestrial plants, to be the most accurate. This model helped us discover that water hyacinth is very efficient at using light when it is dim and can safely reduce damage when light is too strong. The plant achieves this by having a large number of special molecules that absorb light and smartly managing how energy is used or released. These features explain how the plant grows so successfully in different water environments, often outcompeting native species. Our results provide new ways to understand and predict how aquatic plants grow, which can help manage harmful plant invasions and improve the health of water ecosystems.

## 1. Introduction

Aquatic macrophytes play pivotal roles in ecosystem functioning by mediating biogeochemical cycles and creating habitat heterogeneity within aquatic environments [1]. However, their photosynthetic traits fundamentally differ from terrestrial plants due to the unique environmental constraints, including rapid light attenuation in water [2], thermal fluctuations [3], and variable nutrient bioavailability [4]. Floating macrophytes such as *Pontederia crassipes* (Mart.) Solms (water hyacinth) face a unique set of challenges at the air–water interface. Their leaves are exposed to intensified light regimes, which combine direct sunlight and amplified irradiance from water surface reflection, increasing total light exposure by 5–15% depending on solar angle and wave conditions [5]. Concurrently, they contend with self-shading within dense populations and shading from adjacent shoreline, riparian, and emergent vegetation [2]. This dual pressure necessitates adaptive strategies to balance photoprotection against intense sunlight with efficient light harvesting under low-light conditions, a dynamic that is critical to their ecological success.

*P. crassipes*, a free-floating perennial monocot native to South America, exemplifies a striking ecological paradox. Introduced in China as an ornamental species in the early 20th century, it has since become a pervasive invasive plant in ponds, reservoirs, and rivers [1]. Notorious for its rapid clonal growth and ramet-driven vegetative reproduction that disrupts aquatic ecosystems, *P. crassipes* exhibits extraordinary physiological plasticity, enabling tolerance to a wide range of environmental stressors [6,7]. Central to this adaptability is its sophisticated photosynthetic apparatus, which integrates morphological traits (e.g., large floating leaves, vertical petioles, etc.) and biochemical adjustments to optimize light capture and carbon assimilation [7,8].

Plant leaves absorb, excite, transmit, and convert light energy based on the intrinsic properties of their light-harvesting pigments, including spatial structure and charge distribution [9]. The ecological dominance of *P. crassipes* appears closely associated with its ability to regulate photosynthesis [7], including strategies to tolerate or avoid light stress through increased leaf area, optimized photosynthetic efficiency via chloroplast arrangement and stomatal regulation, and vertical growth for enhanced light capture [7,10,11]. Under high light, its glossy leaves minimize photodamage through reflective surfaces and thermal dissipation mechanisms, while in shaded conditions, increased pigment density and chloroplast reconfiguration enhance light absorption efficiency [7,9]. These adaptations are governed by photochemical processes in photosystem II (PSII), where spatial organization of pigments and charge separation dynamics in reaction centers determine the quantum efficiency of electron transport [12,13]. Chlorophyll fluorescence analysis, a non-invasive method for probing PSII dynamics [14], has proven instrumental in characterizing these mechanisms in terrestrial plants. Recent advances integrating gas-exchange, chlorophyll fluorescence, and photosynthetic modeling have clarified photoadaptive strategies in terrestrial systems by precisely characterizing photosynthetic capacity, electron transport dynamics, and PSII down-regulation [15,16,17,18]. However, analogous studies on aquatic macrophytes like *P. crassipes* are lacking, hindering our understanding of their unique photoadaptive physiology.

While terrestrial plants prioritize sustained light utilization, aquatic species like *P. crassipes* must balance efficient low-light harvesting with robust photoprotection. Previous studies highlight its capacity to maintain high photosynthetic efficiency across light gradients [7,10,19], yet the photophysical mechanisms enabling this plasticity remain unresolved. This study presents a comprehensive analysis of photosynthetic performance in *P. crassipes* using simultaneous measurements of chlorophyll fluorescence and gas-exchange parameters using LI-6400 portable photosynthesis system. The purpose is to (a) quantify key photosynthetic light response characteristics; (b) evaluate the applicability of three established photosynthetic models (rectangular hyperbola, non-rectangular hyperbola, and mechanistic models) for aquatic plants; and (c) investigate physiological foundation for its invasive success. Our findings provide novel insights into the photobiological adaptations of aquatic macrophytes and establish methodological frameworks for analyzing aquatic photosynthesis.

## 2. Materials and Methods

### 2.1. Plant Material

Mature *P. crassipes* plants were used for the experiment. They grew naturally in a eutrophic pond adjacent to Jinggangshan University, Ji’an City, Jiangxi Province, China (27.09° N, 115.03° E; elevation 381.6 m). Table 1 provides the nutrient levels of the pond water from our previous study [20]. The plants were in a phase of vigorous vegetative growth, reaching heights of 31–52 cm and displaying 5–8 leaves with well-developed root system.

### 2.2. Gas-Exchange and Chlorophyll Fluorescence Measurements

Measurements were taken on clear days in July 2019, from 8:30–11:30 and 14:00–17:30, at an average daytime temperature of 36 °C. In this region, photosynthetically active radiation (PAR) typically reached around 2200 μmol photons m^−2^ s^−1^ in summer. Five to seven biologically independent plants with uniform growth were randomly selected, with fully expanded leaves from the upper canopy designated for measurement.

Prior to measurements, the selected leaves were light-adapted for one hour under natural light to ensure full activation of rubisco and stabilization of stomatal conductance. Simultaneous recordings of chlorophyll fluorescence and gas-exchange parameters were obtained using a LI-6400 portable photosynthesis system equipped with a 6400-40 leaf chamber fluorometer (Li-Cor Inc., Lincoln, NE, USA). The open-path system maintained controlled conditions, including a CO_2_ concentration of 390 μmol mol^−1^, relative humidity of 50–70%, and air temperature within ±1 °C. A 16-step light intensity (*I*) gradient (2400, 2200, 2000, 1800, 1600, 1400, 1200, 1000, 800, 600, 400, 200, 150, 100, 50, and 0 μmol photons m^−2^ s^−1^) was applied using the embedded “Flr Light Curve” automated protocol. Each light step included a 120–180 s equilibration period, followed by automated reference/sample cell matching. Steady-state measurements of net photosynthetic rate (*P*_n_), electron transport rate (*J*), stomatal conductance (*g*_s_), transpiration rate (*T*_r_), intercellular CO_2_ concentration (*C*_i_), PSII quantum efficiency (*Φ*_PSII_), and non-photochemical quenching (*NPQ*) were recorded.

### 2.3. Photosynthesis Models and Calculations

#### 2.3.1. Rectangular Hyperbola (RH) Model

The rectangular hyperbola model [21] is one of the classic models used to describe the photosynthetic light-response curve of plants. This model is characterized by its simplicity, minimal parameter requirements, and ease of computation. The mathematical representation of the RH model is given by the following:

(1) $$P_{n}=\frac{\alpha IP_{\mathrm{nmax}}}{\alpha I{+P}_{\mathrm{nmax}}}-R_{d}$$

where *P*_nmax_ represents the maximum net photosynthetic rate, *α* is the initial slope of *P*_n_*–I* response curve, and *R*_d_ is the dark respiration rate. Additionally, this model was employed to characterize the response of *J* to *I*.

#### 2.3.2. Non-Rectangular Hyperbola (NRH) Model

The NRH model [22,23] introduces a curvature parameter (*θ*) to correct the “convexity” between low and high light intensities, thereby improving the accuracy of light-response curve fitting. Combined with the Farquhar-von Caemmerer-Berry (FvCB) biochemical model [24], the NRH model has become one of the core frameworks for modeling plant photosynthesis. The relationship between *P*_n_ and *I* in the NRH model is expressed as follows:

(2) $$P_{n}=\frac{\alpha I+P_{\mathrm{nmax}}-\sqrt{(\alpha I+P_{\mathrm{nmax}}{)}^{2}-4\alpha \theta IP_{\mathrm{nmax}}}}{2\theta }-R_{d}$$

where *θ* represents the convexity of curve, and other parameters are as previously defined. Similar to the RH model, this model has also been applied to describe the response of *J* to *I*.

#### 2.3.3. Photosynthetic Mechanistic Model (Ye Model)

The Ye model [25] is a mechanistic representation of the photosynthetic process, explicitly incorporating primary photophysical and photochemical reactions, including light absorption, exciton resonance transfer, quantum energy level transitions, and de-excitation. The model integrates photochemical reactions, exciton transfer, and physiological heat dissipation mechanisms. Through statistical weighting parameters, it quantitatively describes the partitioning of absorbed energy among photochemical reactions, thermal dissipation, and fluorescence. Importantly, this model can directly account for photoinhibition effects and provides an accurate representation of photosynthesis across the entire range of light intensities, from low light to saturation and inhibition [26,27,28]. The mathematical formulation of the Ye model is given as follows:

(3) $$P_{n}=\frac{\alpha^{\prime }\beta^{\prime }N_{0}\sigma_{ik}\varphi \eta }{S}\times \frac{1-\frac{\left(1-\frac{g_{i}}{g_{k}}\right)\sigma_{ik}\tau }{\xi_{3}+\left(\xi_{1}k_{P}+\xi_{2}k_{D}\right)\tau }I}{1+\frac{\left(1+\frac{g_{i}}{g_{k}}\right)\sigma_{ik}\tau }{\xi_{3}+\left(\xi_{1}k_{P}+\xi_{2}k_{D}\right)\tau }I}I-R_{d}$$

where *α*′ represents the light energy distribution coefficient between PSII and PSI (dimensionless), *β*′ is the leaf light absorption coefficient (dimensionless), *N*_0_ is the number of light-harvesting pigment molecules, *σ*_ik_ is the intrinsic light absorption cross-section of light-harvesting pigment molecules (m^2^), *φ* denotes the exciton utilization efficiency (dimensionless), *η* is the efficiency of photosynthetic electron transport (its reciprocal represents the number of electron required to assimilate one CO_2_ molecule), and *S* represents the measured leaf area (m^2^). *g*_i_ and *g*_k_ are the energy level degeneracies of the light-harvesting pigment molecule in ground and excited states, respectively (dimensionless). *ξ*_1_, *ξ*_2_, and *ξ*_3_ are the statistical weighting factors for exciton transfer to photochemistry, heat dissipation, and fluorescence, respectively (dimensionless). *k*_P_ is the photoreaction rate constant (s^−1^). *k*_D_ is the heat dissipation rate constant (s^−1^). *τ* represents the average lifetime of the light-harvesting pigment molecule in the lowest excited state (s^−1^) [25].

According to Equation (3), *P*_n_ depends on multiple biophysical parameters, including *α*′, *β*′, *N*_0_, *σ*_ik_, *φ*, *η*, *S*, *g*_i_, *g*_k_, *ξ*_1_, *ξ*_2_, *ξ*_3_, *k*_P_, *k*_D_, and *τ*. Under steady-state conditions, these parameters are intrinsic to the plant species but may vary under different environmental conditions. To facilitate practical applications, three aggregate parameters are introduced to simplify Equation (3): $\alpha_{p}=\frac{\alpha^{\prime }\beta^{\prime }N_{0}\sigma_{ik}\varphi \eta }{S}$ (μmol electrons (μmol photons)^−1^), $\beta_{p}=\frac{\left(1-\frac{g_{i}}{g_{k}}\right)\sigma_{ik}\tau }{\xi_{3}+\left(\xi_{1}k_{P}+\xi_{2}k_{D}\right)\tau }$ (m^2^ s (μmol photons)^−1^), and $\gamma_{p}=\frac{\left(1+\frac{g_{i}}{g_{k}}\right)\sigma_{ik}\tau }{\xi_{3}+\left(\xi_{1}k_{P}+\xi_{2}k_{D}\right)\tau }$ (m^2^ s (μmol photons)^−1^).

(4) $$P_{n}=\alpha_{p}\frac{1-\beta_{p}I}{1+\gamma_{p}I}I-R_{d}$$

where *α*_p_ is the initial slope of the *P*_n_*–I* response curve, while *β*_p_ and *γ*_p_ are parameters characterizing light limitation and light saturation, respectively.

The photosynthetic quantum efficiency ($P_{n}^{\prime }$), defined as the number of CO_2_ molecules fixed per photon absorbed at a given *I*, is derived from Equation (4) as:

(5) $$P_{n}^{\prime }=\frac{\gamma_{p}-\alpha_{p}I^{2}}{{\left(\gamma_{p}+\beta_{p}I+\alpha_{p}I^{2}\right)}^{2}}$$

The maximum *P*_n_ (*P*_nmax_) is determined by the following:

(6) $$P_{\mathrm{nmax}}=\alpha_{p}{\left(\frac{\sqrt{\beta_{p}+\gamma_{p}}-\sqrt{\beta_{p}}}{\gamma_{p}}\right)}^{2}-R_{d}$$

While the saturation *I* (*I*_sat_) is given by the following:

(7) Isat=(βp+γp)βp−1γp

Ye et al. [25] also established the relationship between *J* and *I* in their photosynthetic mechanistic model using the following equation:

(8) $$J=\alpha_{e}\frac{1-\beta_{e}I}{1+\gamma_{e}I}I$$

where *α*_e_ is the initial slope of the *J–I* response curve, *β*_e_ is PSII dynamics down-regulation coefficient, and *γ*_e_ is the saturation coefficient.

The maximum *J* (*J*_max_) is determined by the following:

(9) $$J_{\max}=\alpha_{e}{\left(\frac{\sqrt{\beta_{e}+\gamma_{e}}-\sqrt{\beta_{e}}}{\gamma_{e}}\right)}^{2}$$

while the saturation *I* (*I*_e-sat_) is given by the following:

(10) Ie-sat=(βe+γe)βe−1γe

Additionally, by incorporating chlorophyll content (unit: mg m^−2^), Equation (8) can be used to simulate *J–I* curves, allowing the extraction of key traits characterizing light-harvesting pigment molecules, including the total photosynthetic pigment molecules (*N*_0_), the eigen-absorption cross-section of photosynthetic pigment molecules (*σ*_ik_), the minimum average lifetime of the lowest excited-state photosynthetic pigment molecules (*τ*_min_), the effective absorption cross-section of pigment molecules (*σ′*_ik_), and the total excited-state pigment molecules (*N*_k_). Moreover, building upon the *P*_n_-*I* and *J-I* mechanistic models, Ye and Yang et al. developed quantitative models describing the light response of light-use efficiency (*LUE*), carboxylation efficiency (*CE*), intrinsic and instantaneous *WUE* (*WUE*_i_ and *WUE*_inst_, respectively) [17], *Φ*_PSII_, and *NPQ* [18]. These interconnected models provide a comprehensive framework for quantifying plant photosynthetic physiology, as detailed previously.

### 2.4. Statistical Analysis

Non-linear regression was performed to fit *P*_n_–*I*, *J–I*, *NPQ*–*I*, *Φ*_PSII_–*I*, *LUE–I*, *CE–I*, *WUE*_i_–*I*, and *WUE*_inst_–*I* curves using the *Photosynthesis Model Simulation Software* (PMSS, Jinggangshan University) (http://photosynthetic.sinaapp.com, in Chinese/English version, accessed on 5 March 2025). The goodness of fit of the three models was evaluated by the coefficient of determination (*R*^2^ = 1−SSE/SST, where SST is the total sum of squares, and SSE is the error sum of squares), Akaike’s information criterion (*AIC*), and mean absolute error (MAE = $\frac{1}{n}\sum_{i=1}^{n}\left|\hat{y_{i}}-y_{i}\right|$, where $\hat{y_{i}}$ is the fitted values from model, and $y_{i}$ is the measured values). A one-way analysis of variance (ANOVA) was conducted using SPSS Statistics 24.0 to compare differences between the model-fitted and measured values, with statistical significance set at *p* < 0.05. The ratio of *J*_max_ to *P*_nmax_ can be used to estimate the apparent number of electrons required to assimilate one molecule of CO_2_ (*n*_a_), providing an indicator of the photosynthetic electron utilization efficiency in plant leaves. Data are presented as mean ± *SE* (*n* = 4).

## 3. Results

### 3.1. Photosynthetic and Electron Transport Responses

**Figure 1:**
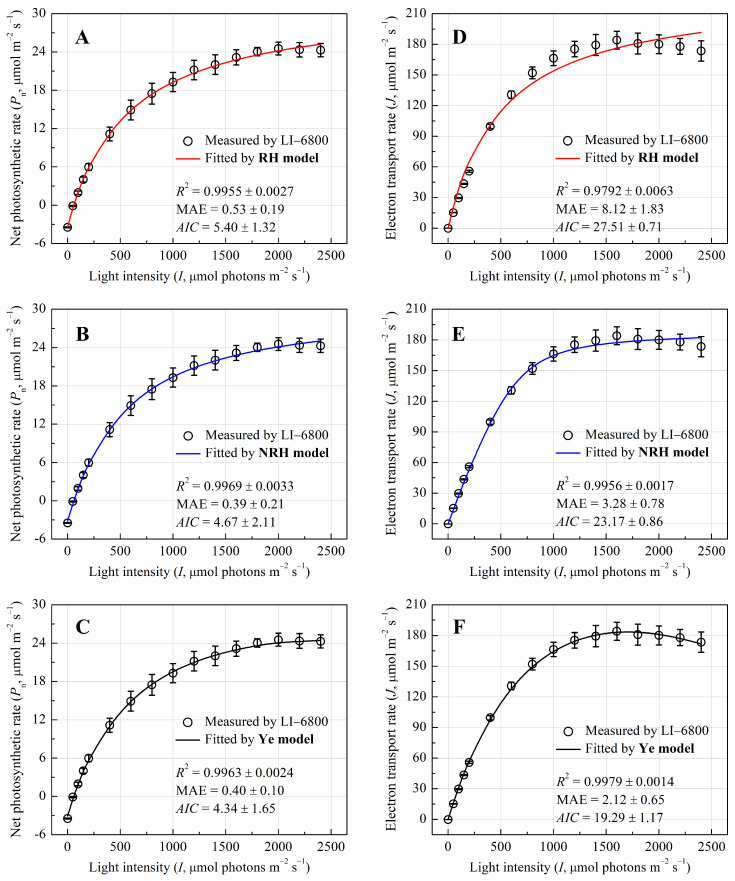
Light-response curves of the net photosynthetic rate (*P*_n_) and electron transport rate (*J*) for *Pontederia crassipes*. The curves were simulated by RH model (**A**,**D**), NRH model (**B**,**E**), and Ye model (**C**,**F**), respectively.

The photosynthetic light-response dynamics of *P. crassipes* were rigorously assessed through coupled chlorophyll fluorescence and gas-exchange analyses. Figure 1A–C illustrate the light-dependent progression of net photosynthetic rate (*P*_n_), which exhibited a hyperbolic increase with increasing *I*, saturating at approximately 2000 μmol photons m^−2^ s^−1^ without photoinhibition. The Ye mechanistic model demonstrated superior goodness-of-fit (*R*^2^ = 0.9963, MAE = 0.40, *AIC* = 4.34) in replicating the *P*_n_–*I* curve (Figure 1C), with no statistically significant deviation between modeled and observed *P*_nmax_ (24.64 ± 1.08 vs. 24.70 ± 1.01 μmol CO_2_ m^−2^ s^−1^, *p* > 0.05, Table 1). In contrast, both rectangular (RH) and non-rectangular hyperbola (NRH) models overestimated *P*_nmax_ by 45.7% and 36.4%, respectively (*p* < 0.05), and failed to yield saturation light intensity (*I*_sat_), highlighting their limitations in capturing high-light dynamics (Figure 1A,B; Table 2).

Parallel analysis of electron transport rate (*J*) revealed a biphasic response to *I*, peaking at 186.07 ± 10.04 μmol CO_2_ m^−2^ s^−1^, and then, it gradually declined, indicating dynamic down-regulation of PSII activity (Figure 1D–F). The Ye model precisely simulated this trajectory (*R*^2^ = 0.9979, MAE = 2.12, *AIC* = 19.29), whereas RH and NRH models diverged markedly at supra-optimal light intensity, overestimating *J*_max_ by 24.7% and 1.5%, respectively (Table 2). This also led to a significant overestimation of the value of apparent electron requirement for CO_2_ assimilation (*n*_a_) in both models. However, the *n*_a_ value from the Ye model closely aligned with the measured value (Table 2).

### 3.2. Quantum Yield and Photophysical Traits of Light-Harvesting Pigment Molecules

**Figure 2:**
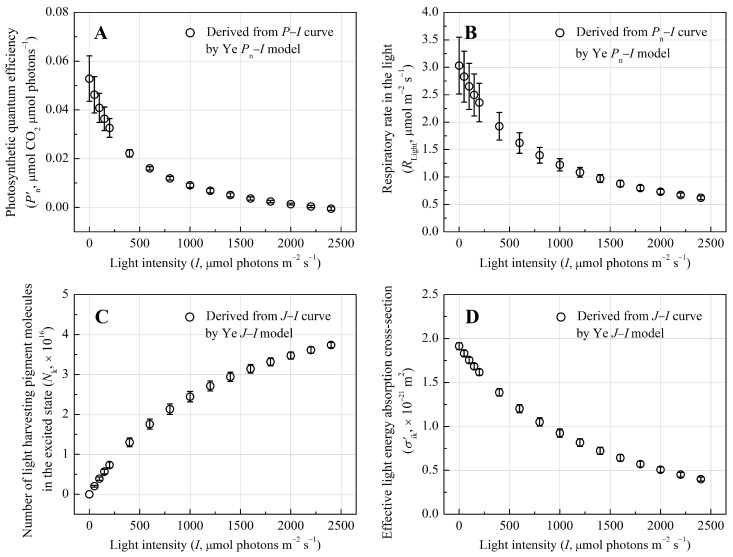
Ligh-response curves of the photosynthetic quantum efficiency ($P_{n}^{\prime }$, (**A**)), respiratory rate in the light (*R*_Light_, (**B**)), total excited-state pigment molecules (*N*_k_, (**C**)), and effective absorption cross-section (*σ′*_ik_, (**D**)) for *Pontederia crassipes*.

Photosynthetic quantum efficiency ($P_{n}^{\prime }$), reflecting the efficiency of light energy conversion, decreased from 0.023 ± 0.002 to −0.47 × 10^−3^ μmol CO_2_ μmol photons^−1^ with increasing *I*, indicating diminished light utilization (Figure 2A). The negative $P_{n}^{\prime }$ at *I* = 2400 μmol photons m^−2^ s^−1^ indicates that respiratory CO_2_ release exceeded photosynthetic CO_2_ fixation, which was supported by the stabilization of respiratory rate in the light (*R*_Light_) at 2.97% of *P*_n_ under saturating light (Figure 2B). Mechanistic modeling of *J*–*I* curves resolved intrinsic photophysical parameters of light-harvesting pigments. As shown in Table 2, the total photosynthetic pigment pool (*N*_0_ = 9.46 ± 0.08 × 10^16^) and eigen-absorption cross-section (*σ*_ik_ = 1.91 ± 0.04 × 10^−21^ m^2^) underscored robust light-capturing capacity. The minimum average lifetime of the lowest excited-state pigment molecules (*τ*_min_) was 11.53 ± 1.27 ms. The total excited-state pigment molecules (*N*_k_) increased with *I* (Figure 2C), suggesting a gradual shift toward energy dissipation rather than abrupt saturation. Despite a 73.8% reduction in effective absorption cross-section (*σ′*_ik_) under high light (0.5 × 10^−21^ m^2^ at *I* = 2000 μmol photons m^−2^ s^−1^, Figure 2D), the residual absorption capacity remained sufficient to sustain photosynthetic activity, reflecting adaptive plasticity in light harvesting.

### 3.3. Photoprotection and Metabolic Efficiency Dynamics

**Figure 3:**
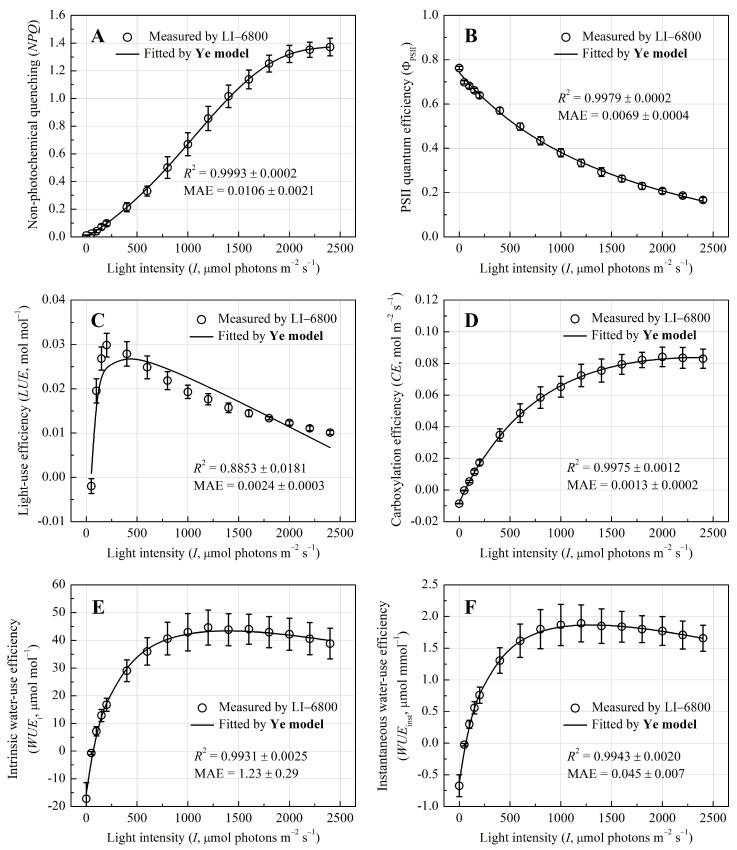
Light-response curves of the non-photochemical quenching (*NPQ*, (**A**)), PSII quantum efficiency (Φ_PSII_, (**B**)), light-use efficiency (*LUE*, (**C**)), carboxylation efficiency (*CE*, (**D**)), intrinsic water-use efficiency (*WUE*_i_, (**E**)), and instantaneous *WUE* (*WUE*_inst_, (**F**)) for *Pontederia crassipes*.

Non-photochemical quenching (*NPQ*) increased monotonically to 1.375 ± 0.062 at *I* = 2350 μmol photons m^−2^ s^−1^ (Figure 3A), consistent with enhanced thermal dissipation under high light. This photoprotective response coincided with a decline in PSII quantum efficiency (Φ_PSII_) from 0.762 ± 0.007 to 0.412 ± 0.011 (Figure 3B), suggesting reduced photochemical efficiency as light saturation intensified. Light-use efficiency (*LUE*) followed a typical peaked response, with a sharp rise at low light and a gradual decline beyond saturation at approximately 200 μmol photons m^−2^ s^−1^ (Figure 3C), while carboxylation efficiency (*CE*) reached its maximum 0.085 mol m^−2^ s^−1^ at *I* = 2200 μmol photons m^−2^ s^−1^ (Figure 3D), reflecting distinct thresholds for light capture and CO_2_ assimilation. Water-use metrics exhibited analogous saturation patterns, with intrinsic water-use efficiency (*WUE*_i_) plateauing at *I* = 1600 μmol photons m^−2^ s^−1^ (45.91 ± 6.28 μmol mol^−1^, Figure 3E) and instantaneous *WUE* (*WUE*_inst_) stabilizing earlier at *I* = 1400 μmol photons m^−2^ s^−1^ (1.96 ± 0.29 mmol^−1^, Figure 3F).

Critically, derivative models from the Ye mechanism framework (e.g., *NPQ*–*I*, Φ_PSII_–*I*, *CE–I*, *WUE*_i_–*I*, *WUE*_inst_–*I*) accurately simulated photoprotective and metabolic responses (*R*^2^ > 0.99) (Figure 3). Fitted *NPQ*_max_, *CE*_max_, *WUE*_i-max_, and *WUE*_inst-max_ and their saturation light intensities closely matched the measured values (*p* > 0.05) (Table 3). While the Φ_PSII_–*I* model slightly underestimated Φ_PSII-max_, the *LUE*–*I* model only moderately captured *LUE* changes, exhibiting larger discrepancies compared to other parameters.

## 4. Discussion

### 4.1. Applicability of Ye Mechanistic Model in Aquatic Plants Photosynthesis

Our results demonstrate the superior performance of Ye mechanistic model in the simulating photosynthetic light-response curves of *P. crassipes* compared to traditional empirical models (RH, NRH), aligning with previous studies that have validated the Ye model in various plant species, including terrestrial plants [26,27,28,29] and cyanobacteria [30]. The Ye model’s advantage lies in its detailed incorporation of both photophysical and photochemical processes. Specifically, it accounts for light absorption (*σ*_ik_), exciton transfer efficiency (*φ*), and energy dissipation dynamics (*k*_D_, *τ*), all critical for accurately representing aquatic photosynthesis in variable light conditions.

While the RH and NRH models provided reasonable approximations under low light, they fell short in capturing high-light saturation and the dynamic down-regulation of PSII. These models overestimated the *P*_nmax_ by 36–46% and the *J*_max_ by 1.5–24.7% because they assume linear or hyperbolic light responses without accounting for the underlying physiological mechanisms of photosynthesis [17]. These findings align with prior critiques of empirical models in terrestrial systems [17,31,32,33], which lack the flexibility to simulate dynamic photoinhibition or species-specific photoprotective strategies. In contrast, the Ye model’s parameterization of *N*_k_ (total excited-state pigments), *τ*_min_ (the minimum average lifetime of excited-state pigments), and *σ*′_ik_ (effective absorption cross-section) explains how *P. crassipes* rapidly dissipates excess energy as heat under high light, thereby avoiding photodamage. In addition, the close alignment between the modeled and observed *J*_max_/*P*_nmax_ ratio (*n*_a_ = 7.46 vs. 7.52) in *P. crassipes* underscores the Ye model’s ability to quantify electron transport efficiency. This ratio reflects the balance between linear electron flow (driven by PSII activity) and carboxylation efficiency, a relationship inherently modulated by large *N*_0_ and adaptive *NPQ* [34]. Traditional models, which oversimplify electron transport as a static function of light, fail to resolve these interdependencies. The Ye model’s mechanistic basis thus provides a critical tool for studying aquatic plants, where environmental variability (e.g., light fluctuations, nutrient gradients, etc.) demands precise representation of energy allocation and stress responses.

### 4.2. Evolutionary Adaptations of P. crassipes

The photosynthetic performance of *P. crassipes* reveals evolutionary adaptations that differentiate it from native aquatic macrophytes. As shown in Table 4, its *P*_nmax_ and *I*_sat_ exceed values reported for other aquatic plants like *Nymphoides peltate*, *Nelumbo nucifera*, and *Phragmites australis* [35,36,37], which show *P*_nmax_ values < 20 μmol CO_2_ m^−2^ s^−1^ and earlier photoinhibition thresholds. Even its photosynthetic capacity exceeds that of some common C_3_ plants such as *Oryza sativa* [38], *Tamarix ramosissima* [39], *Solanum lycopersicum* L. [40], *Malus pumila* Mill. [41], and *Glycine max* L. (Merr.) [30]. The *I*_sat_ is close to C_4_ maize [19] and exceeds C_4_ sorghum [17]. These traits suggest that *P. crassipes* has evolved more efficient mechanisms for light absorption and energy dissipation, making it particularly competitive in nutrient-rich, high-light environments. The plant’s large photosynthetic pigment pool (9.46 × 10^16^ molecules m^−2^) and high eigen-absorption cross-section (1.91 × 10^−21^ m^2^) enhance photon-absorption efficiency, which is coupled with dynamic photoprotective responses, such as *NPQ*, to prevent photodamage under high light.

This high photosynthetic rate drives rapid growth and clonal propagation in *P. crassipes* [42]. The clonal growth strategy of *P. crassipes* is achieved through efficient allocation of photosynthetic carbon. Studies have shown that the mother plant preferentially supports stolon extension and daughter plant formation [42,43,44]. Under high CO₂ conditions, its high photosynthetic rate enables a single plant to produce 3000 new individuals within 50 days [43]. Furthermore, its high *I*_sat_ and quantum efficiency (*α*_p_ = 0.0528) ensure sustained photosynthesis and ramet production under high light [19,44]. The case in Portugal shows that high light intensity is highly consistent with the seasonal expansion of ramet coverage area [45]. Therefore, the invasion success of *P. crassipes* is essentially the result of the synergistic effect of its efficient photosynthesis and clonal reproduction strategy [42,43,44]. Ramet production not only relies on direct support from photosynthetic products, but also strengthens population competitiveness by changing niche and diffusion patterns.

Interestingly, regional variation in photosynthetic capacity, such as the higher *P*_nmax_ observed in *P. crassipes* populations in Nanjing, China (32.03° N, 118.88° E; elevation 10 m) [19] and in the Federal University of Lavras, state of Minas Gerais, Brazil (19.913° S, 43.941° W; elevation 830 m) [7], indicates the species’ ability to adapt to local light conditions. This adaptability contrasts sharply with native species like *N. peltate* [35], which exhibit more rigid physiological responses to environmental gradients, further emphasizing the ecological flexibility of *P. crassipes*.

### 4.3. Synergistic Efficiency Metrics Supports Invasiveness of P. crassipes

The integration of key efficiency metrics—light-use efficiency (*LUE*), water-use efficiency (*WUE*), and carboxylation efficiency (*CE*)—provides a comprehensive physiological framework for understanding the invasiveness of *P. crassipes*. The species’ high *LUE*_max_ (0.030 mol mol^−1^) at low light intensity (approximately 200 µmol photons m^−2^ s^−1^), coupled with a sharp decline in *LUE* at higher light levels, indicates its strategic shift toward photoprotection rather than carbon fixation as light intensity increases. This plasticity enables *P. crassipes* to thrive under variable light conditions while maintaining efficient resource utilization. Moreover, the species exhibits superior *WUE*, as reflected in both intrinsic *WUE* (*WUE*_i_) and instantaneous *WUE* (*WUE*_inst_). These metrics suggest that *P. crassipes* effectively regulates stomatal conductance to balance CO_2_ uptake and water conservation, a critical strategy for survival in eutrophic environments. This efficient use of light and water, combined with the species’ high *J*_max_, places *P. crassipes* in direct competition with native macrophytes like *P. australis*, which exhibit lower *CE* and less effective photoprotective mechanisms [37]. However, under high light (>1600 μmol photons m^−2^ s^−1^), as observed in most C_3_ plants, *P. crassipes* also exhibits significant PSII dynamic down-regulation [18].

## 5. Conclusions

In conclusion, this study highlights the utility of the Ye mechanistic model in elucidating the complex photobiological adaptations that contribute to the ecological success of *P. crassipes*. By integrating chlorophyll fluorescence and gas-exchange data, we provide a holistic view of the species’ photosynthetic performance, linking molecular-level mechanisms to its ecosystem-level invasiveness. Our findings demonstrate that the invasive success of *P. crassipes* is underpinned by its exceptional photosynthetic plasticity, including high light-use efficiency under low irradiance, strong photoprotection via elevated *NPQ* under high light, and a large pigment pool enabling broad spectral absorption. These physiological traits allow the plant to sustain carbon assimilation and energy balance across a wide range of environmental conditions, thereby supporting rapid clonal growth and spatial expansion. While vegetative reproduction is a key driver of its proliferation, we also recognize that submerged seedlings exhibit remarkable low-light adaptability, facilitating early-stage competition with native macrophytes in shaded or turbid habitats. Together, these mechanisms explain its capacity to colonize and dominate eutrophic aquatic systems. Future research should extend this approach to other aquatic macrophytes, particularly invasive species, to identify universal physiological traits that predict ecological success. Additionally, comparative studies on life-stage-specific adaptations (e.g., submerged seedlings vs. floating adults) could refine mechanistic models and inform targeted management strategies, such as shading interventions to suppress seedling establishment or nutrient reduction to limit clonal expansion.

## Figures and Tables

**Table 1:** Water quality characteristics of *Pontederia crassipes* growth pond [20].

	Ammonium Nitrogen (NH_4_^+^-N)	Chemical Oxygen Demand (CODcr)	Total Nitrogen (TN)	Total Phosphorus (TP)	Dissolved Oxygen (DO)	pH Value
**Concentration (mg L^−1^)**	1.80 ± 0.04	17.67 ± 0.98	3.09 ± 0.02	0.36 ± 0.01	4.24 ± 0.07	8.21 ± 0.01

**Table 2:** Fitted and measured values of traits defining *P*_n_–*I* and *J*–*I* curves of *Pontederia crassipes*. Values are means ± *SE* (*n* = 4). Within each row, values with different letters are significantly different (*p* < 0.05).

Traits	Fitted Value			Measured Value
	RH Model	NRH Model	Ye Model	
*α*_p_ (μmol mol^−1^)	0.0659 ± 0.0111 a	0.0522 ± 0.0058 a	0.0528 ± 0.0107 a	―
*P*_nmax_ (μmol m^−2^ s^−1^)	35.93 ± 1.33 a	33.70 ± 1.65 a	24.64 ± 1.08 b	24.70 ± 1.01 b
*I*_sat_ (μmol m^−2^ s^−1^)	―	―	2520.41 ± 243.03 a	2200.00 ± 81.65 a
*I*_c_ (μmol m^−2^ s^−1^)	58.78 ± 3.59 a	60.02 ± 3.33 a	54.17 ± 2.45 a	53.06 ± 2.11 a
*R*_d_ (μmol m^−2^ s^−1^)	3.41 ± 0.46 a	2.91 ± 0.24 b	3.46 ± 0.16 a	3.46 ± 0.06 a
*α*_e_ (μmol mol^−1^)	0.4658 ± 0.0109 a	0.2779 ± 0.0052 c	0.3424 ± 0.0076 b	―
*J*_max_ (μmol m^−2^ s^−1^)	232.08 ± 16.78 a	188.85 ± 11.66 b	184.10 ± 10.84 b	186.07 ± 10.04 b
*I*_e-sat_ (μmol m^−2^ s^−1^)	―	―	1699.64 ± 40.39 a	1750.00 ± 170.78 a
*n*_a_	6.45 ± 0.34 b	5.62 ± 0.31 b	7.46 ± 0.12 a	7.52 ± 0.12 a
*σ*_ik_ (10^−21^ m^2^)	―	―	1.91 ± 0.04	―
*τ*_min_ (ms)	―	―	11.53 ± 1.27	―
*N*_0_ (10^16^ m^2^)	―	―	9.46 ± 0.08	―
*Chl* content (mg m^−2^)	―	―	―	707.34 ± 5.86

**Table 3:** Fitted and measured values of photoprotection and physiological traits of *Pontederia crassipes*. Values are means ± *SE* (*n* = 4). Within each row, values with different letters are significantly different (*p* < 0.05).

Traits	Fitted Value	Measured Value
*NPQ*_max_	1.366 ± 0.058 a	1.375 ± 0.062 a
*I*_NPQ-sat_ (μmol m^−2^ s^−1^)	2278.76 ± 41.25 a	2350.00 ± 50.00 a
Φ_PSIImax_	0.743 ± 0.007 b	0.762 ± 0.007 a
*LUE*_max_ (mol mol^−1^)	0.027 ± 0.002 a	0.030 ± 0.003 a
*I*_LUE-sat_ (μmol m^−2^ s^−1^)	384.65 ± 17.60 a	250.00 ± 50.00 b
*CE*_max_ (mol m^−2^ s^−1^)	0.084 ± 0.006 a	0.085 ± 0.006 a
*I*_CE-sat_ (μmol m^−2^ s^−1^)	2242.99 ± 82.55 a	2000.00 ± 81.65 b
*WUE*_i-max_ (μmol mol^−1^)	44.17 ± 6.09 a	45.91 ± 6.28 a
*I*_i-sat_ (μmol m^−2^ s^−1^)	1621.82 ± 267.39 a	1500.00 ± 173.21 a
*WUE*_inst-max_ (μmol mmol^−1^)	1.88 ± 0.28 a	1.96 ± 0.29 a
*I*_inst-sat_ (μmol m^−2^ s^−1^)	1391.42 ± 139.03 a	1300.00 ± 173.21 a

**Table 4:** *Pontederia crassipes* compared to other plants in photosynthetic ability.

Plants	*P*_nmax_	*I*_sat_	*J*_max_	*I*_e-sat_	Reference
*P. crassipes*	23.1–30.8	―	―	―	[7]
*P. crassipes*	34.5 ± 0.72	2358 ± 69	―	―	[19]
*P. crassipes*	24.70 ± 1.01	2200.0 ± 81.7	186.1 ± 10.0	1750.0 ± 170.8	This study
*Nymphoides peltate*	12.66	219.98	―	―	[35]
*Nelumbo nucifera*	7.1–9.2	―	―	―	[36]
*Phragmites australis*	9.0~19.5	924.1–2186.3	―	―	[37]
*Oryza sativa* L.	17.51–27.89	≈2000	―	―	[38]
*Oryza sativa* L. (Kitaake)	19.56 ± 0.62	1641 ± 32.0	―	―	[19]
*Tamarix ramosissima*	17.2–24.4	957–1360	―	―	[39]
*Solanum lycopersicum* L.	6.34–17.82	―	―	―	[40]
*Malus pumila* Mill.	15.25–20.29	1413.8–1874.9	―	―	[41]
*Glycine max* L. (Merr.)	19.73	1800	143.51 ± 5.21	1601.6 ± 0.64	[30]
*Zea mays* L. (Nongda 108)	30.36 ± 0.42	2550 ± 37.0	―	―	[19]
*Sorghum bicolor* L. (KFJT-4)	37.49 ± 0.90	1866.7 ± 33.3	170.15 ± 4.45	1640.0 ± 74.83	[17,18]
*Sorghum bicolor* L. (KFJT-1)	―	―	133.84 ± 5.52	1600.0 ± 63.24	[18]

## Data Availability

Raw measurement data are available in Supplementary Table S1.

## Supplementary Materials

The following supporting information can be downloaded at https://www.mdpi.com/article/10.3390/biology14060600/s1: Table S1. Gas-exchange measurement data.
