# Supplementary material for: Mechanistic Modeling Reveals Adaptive Photosynthetic Strategies of Pontederia crassipes: Implications for Aquatic Plant Physiology and Invasion Dynamics
Source: Biology (Basel). 2025 May 25;14(6):600. doi: 10.3390/biology14060600 (PMC12189231; doi:10.3390/biology14060600)
Supplement: Supplementary file 1 [file biology-14-00600-s001.zip › biology-3629909-supplementary.pdf]

Supplementary Materials

Mechanistic Modeling Reveals Adaptive Photosynthetic Strategies of *Pontederia crassipes*: Implications for Aquatic Plant Physiology and Invasion Dynamics

Lihua Liu [1,†], Xiaolong Yang [2,3,†], Piotr Robakowski [4], Zipiao Ye [5,*,‡], Fubiao Wang [5] and Shuangxi Zhou [6,*]

1 College of Safety Engineering and Emergency Management, Nantong Institute of Technology, Nantong 226002, P. R. of China
2 School of Life Sciences, Nantong University, Nantong 226019, P. R. of China
3 State Key Laboratory of Environmental Chemistry and Ecotoxicology, Research Center for Eco-Environmental Sciences, Chinese Academy of Sciences, Beijing 100085, P. R. of China
4 Department of Forestry, Poznan University of Life Sciences, Wojska Polskiego 71E St., 60–625 Poznan, Poland
5 Math & Physics College, Jinggangshan University, Ji'an 343009, P. R. of China
6 Department of Biological Sciences, Macquarie University, Sydney, NSW, Australia
* Correspondence: yezp@jgsu.edu.cn (Z.Y.); shuangxi.zhou@dpird.wa.gov.au (S.Z.)
† These authors equally contribute to this work.
‡ Current Affiliation: New Quality Productivity Research Center, Guangdong ATV College of Performing Arts, Deqing 526631, P. R. of China.

Table S1 Gas exchange measurement data.

PARi: photosynthetically active radiation, μmol m^{-2} s^{-1}; Photo: net photosynthetic rate, μmol m^{-2} s^{-1}; Ci: intercellular CO_2 concentration, μmol mol^{-1}; Cond: stomatal conductance, mol m^{-2} s^{-1}; *NPQ*: non-photochemical quenching; *ETR*: electron transport rate, μmol m^{-2} s^{-1}; Tr: transpiration rate, mmol m^{-2} s^{-1}; PhiPS2: PSII quantum efficiency, T: air temperature in leaf chamber, °C, and CO_2: air CO_2 concentration in leaf chamber, μmol mol^{-1}.

**Plant No.**	**Gas exchange parameters**									
**1**	**PARi**	**Photo**	C_i	**Cond**	***NPQ***	***ETR***	T_r	**PhiPS2**	**T**	CO_2
	**in**	**out**	**out**	**out**	**out**	**out**	**out**	**out**	**in**	**in**
	2385.728	26.03255	276.528	0.508781	1.336539	176.1054	13.83069	0.16895	36.38	389.62
	2184.688	26.02816	274.7387	0.502032	1.296391	181.544	13.60502	0.190114	36.56	388.77
	1986.689	25.66217	275.8318	0.496258	1.253067	182.3256	13.33808	0.209856	36.64	389.32
	1799.183	25.24711	276.1964	0.493126	1.160621	187.7198	13.06782	0.238487	36.64	388.37
	1600.789	25.43792	276.119	0.488379	1.005613	189.3213	12.61487	0.270334	36.49	389.41
	1399.469	24.77334	277.3701	0.48151	0.853967	193.5812	12.14813	0.316181	36.34	388.89
	1198.244	24.07432	278.872	0.467438	0.670608	186.8074	11.60475	0.35636	36.20	389.84
	1000.749	22.32771	284.7343	0.462092	0.496499	175.6353	11.10328	0.401159	35.95	389.25
	800.7225	20.7679	286.1197	0.432148	0.350086	160.4771	10.29275	0.458129	35.75	389.13
	598.6152	18.12757	293.3146	0.401527	0.264047	134.9324	9.425992	0.515369	35.49	390.06
	398.7994	13.34656	307.0689	0.351891	0.167441	101.4103	8.333528	0.581332	35.33	389.85
	199.5689	6.476614	337.2827	0.310025	0.076312	55.78155	7.297688	0.639159	35.04	389.39
	151.1056	4.226846	341.9987	0.234706	0.054144	43.78219	5.904153	0.662529	35.13	389.02
	99.32911	1.916984	352.6532	0.167871	0.028658	29.69417	4.574225	0.683463	35.38	389.07
	48.46996	-0.27237	375.1095	0.123677	0.013203	14.86402	3.556385	0.700841	35.53	389.78
	-0.25573	-3.58588	426.1942	0.1048	0.007414	-0.08393	3.054794	0.754483	35.52	389.18
**3**	**PARi**	**Photo**	C_i	**Cond**	***NPQ***	***ETR***	T_r	**PhiPS2**	**T**	CO_2
	**in**	**out**	**out**	**out**	**out**	**out**	**out**	**out**	**in**	**in**
	2385.872	26.15917	281.5073	0.591534	1.270044	203.9978	12.33797	0.195687	34.38	389.78
	2188.309	26.6008	279.5408	0.552006	1.280958	201.3716	11.90843	0.21052	35.27	388.33

1989.379	26.81149	278.4448	0.525337	1.224779	209.4366	11.44165	0.240725	35.77	389.53
1799.715	25.12113	280.6258	0.495401	1.164053	211.0721	10.74343	0.268071	35.90	389.08
1600.649	24.88154	280.729	0.471212	1.056667	210.445	10.18525	0.300522	35.99	388.31
1399.93	24.28895	279.9074	0.442523	0.917053	205.6184	9.589426	0.335736	36.08	390.22
1199.817	22.85232	279.467	0.392234	0.753332	193.8974	8.67494	0.369387	36.19	389.35
1000.966	20.74286	280.8755	0.351534	0.567804	183.9131	7.611523	0.420057	35.85	389.44
801.3071	18.84954	285.8445	0.355395	0.393028	165.8118	7.344151	0.473096	35.38	388.47
600.7985	15.55887	297.9397	0.354325	0.274153	139.5865	7.136104	0.53119	34.81	389.12
399.4883	11.10228	316.3836	0.33384	0.150032	105.4947	6.576514	0.603671	34.16	388.04
201.6812	6.809194	340.5893	0.329652	0.061632	58.77538	6.473666	0.666409	33.83	390.14
149.0361	4.699682	350.204	0.314186	0.035685	44.59274	6.35887	0.684076	33.75	389.98
100.39	2.478702	361.5676	0.297332	0.015506	30.94045	6.23123	0.704623	33.80	390.15
48.81391	0.099621	374.9153	0.283304	0.007318	15.2347	6.060797	0.713257	33.85	389.89
-0.02793	-3.30319	396.3554	0.275467	0.003294	-0.00951	5.863613	0.782871	33.72	390.01

**4**	**PARi**	**Photo**	***C_i***	**Cond**	***NPQ***	***ETR***	***T_r***	**PhiPS2**	**T**	**CO_2**
	**in**	**out**	**out**	**out**	**out**	**out**	**out**	**out**	**in**	**in**
	2386.159	23.83227	317.9816	0.868256	1.325342	163.8956	16.60262	0.157203	35.22	388.94
	2188.266	23.52983	318.3212	0.846278	1.318187	159.6855	16.33284	0.166946	35.31	389.76
	1989.008	22.99215	318.9008	0.83665	1.309132	163.9232	16.24227	0.188451	35.45	389.58
	1800.264	22.86249	318.6681	0.836989	1.266851	161.2887	16.14593	0.204782	35.57	388.91
	1599.889	21.67553	314.6977	0.721941	1.181137	170.1971	15.12	0.24316	35.97	389.28
	1399.18	20.7506	315.6721	0.69636	1.079543	160.5386	14.44367	0.262262	35.90	389.17
	1201.85	20.71542	316.2625	0.683416	0.943958	163.1088	13.76487	0.310219	35.59	390.12
	1000.588	18.7783	319.9352	0.651376	0.759548	157.5811	12.93193	0.359973	35.28	390.11
	800.5795	17.18567	321.3037	0.606328	0.575428	144.2068	11.99207	0.411838	35.05	389.63
	599.4964	15.34629	325.5235	0.589512	0.385042	128.8588	11.50439	0.491448	34.97	388.97

	398.8984	12.01752	334.8023	0.566777	0.263307	97.78974	11.04902	0.560438	34.96	388.77
	199.2505	6.204924	353.4753	0.525102	0.131083	55.89236	10.32695	0.641452	34.88	389.56
	150.6854	4.282042	359.5554	0.496774	0.117987	43.59727	10.07897	0.66157	34.99	389.92
	98.7178	2.211417	367.1262	0.487853	0.055904	29.47406	10.02085	0.682598	35.04	390.11
	51.03093	-0.02376	375.8723	0.467505	0.038495	15.65058	9.763374	0.701131	35.03	390.63
	-0.34036	-3.53396	390.395	0.437642	0.019322	-0.11302	9.263359	0.763369	34.94	390.75
**5**	**PARi**	**Photo**	***C_i***	**Cond**	***NPQ***	***ETR***	***T_r***	**PhiPS2**	**T**	**CO_2**
	**in**	**out**	**out**	**out**	**out**	**out**	**out**	**out**	**in**	**in**
	2382.191	22.17652	0.658326	302.5841	1.556315	150.1865	17.58445	0.1443	37.74	389.69
	2185.627	22.26354	0.622202	301.713	1.515229	169.4416	16.92773	0.177367	37.85	389.16
	1985.274	22.74115	0.588547	301.3236	1.50317	164.6536	16.17633	0.189653	37.82	388.84
	1799.643	22.95996	0.539706	300.4894	1.418312	163.1993	15.07979	0.207276	37.70	388.81
	1600.961	20.6483	0.500197	301.669	1.310055	166.6999	14.065	0.238007	37.50	389.72
	1399.63	18.29171	0.466434	302.8005	1.213546	157.8827	13.12662	0.257844	37.29	389.64
	1201.112	17.11254	0.444834	304.9167	1.054724	157.831	12.61293	0.30037	37.26	389.83
	1000.603	15.38936	0.432321	306.9264	0.856216	148.3371	11.98837	0.338858	36.94	390.04
	800.4479	13.10548	0.395847	312.2287	0.685999	137.8213	11.01879	0.393587	36.72	390.12
	601.7759	10.66558	0.369013	320.4251	0.401832	119.5422	10.27439	0.454204	36.57	390.33
	401.7174	8.170406	0.344219	330.5966	0.277643	93.92841	9.613742	0.534559	36.50	391.06
	198.771	4.409814	0.320809	348.8981	0.120781	52.95982	8.789609	0.609262	36.20	391.31
	150.6216	2.896579	0.289079	355.411	0.079587	41.96338	8.005995	0.637046	36.07	391.20
	99.02304	1.210223	0.273207	365.2867	0.060563	28.42046	7.374713	0.656169	35.68	390.89
	51.2309	-0.20044	0.249742	375.3635	0.032841	15.19222	6.573905	0.677941	35.20	391.50
	-0.34657	-3.41646	0.234588	398.1905	0.010505	-0.11292	5.973066	0.749015	34.70	390.37
